# Osteogenic Potential of Magnesium (Mg)-Doped Multicomponent Bioactive Glass: In Vitro and In Vivo Animal Studies

**DOI:** 10.3390/ma15010318

**Published:** 2022-01-03

**Authors:** Saeid Kargozar, Peiman Brouki Milan, Moein Amoupour, Farzad Kermani, Sara Gorgani, Simin Nazarnezhad, Sara Hooshmand, Francesco Baino

**Affiliations:** 1 Tissue Engineering Research Group (TERG), Department of Anatomy and Cell Biology, School of Medicine, Mashhad University of Medical Sciences, Mashhad 917794-8564, Iran; sara.gorgani70n@gmail.com (S.G.); smn.nazarnezhad@yahoo.com (S.N.); 2 Cellular and Molecular Research Centre, Iran University of Medical Sciences, Tehran 144961-4535, Iran; 3 Department of Tissue Engineering and Regenerative Medicine, Faculty of Advanced Technologies in Medicine, Iran University of Medical Sciences, Tehran 144961-4535, Iran; 4 Department of Medical Biotechnology, Faculty of Allied Medicine, Iran University of Medical Sciences, Tehran 144961-4535, Iran; moein.amoupour@gmail.com; 5 Department of Materials Engineering, Faculty of Engineering, Ferdowsi University of Mashhad (FUM), Azadi Sq., Mashhad 917794-8564, Iran; farzadkermani73@gmail.com; 6 Nanotechnology Research and Application Center (SUNUM), Sabanci University, Istanbul 34956, Turkey; sara.houshmand@sabanciuniv.edu; 7 Department of Applied Science and Technology (DISAT), Institute of Materials Physics and Engineering, Politecnico di Torino, 10129 Torino, Italy

**Keywords:** bioactive glasses, scaffold, magnesium, ion release, osteogenesis, bone tissue engineering

## Abstract

The use of bioactive glasses (BGs) has been quite fruitful in hard tissue engineering due to the capability of these materials to bond to living bone. In this work, a melt-derived magnesium (Mg)-doped BG (composition: 45SiO_2_–3P_2_O_5_–26CaO–15Na_2_O–7MgO–4K_2_O (mol.%)) was synthesized for being used in bone reconstruction. The prepared BGs were then manufactured as three-dimensional (3D) scaffolds by using the sponge replica approach. The microstructure of the samples was assessed by X-ray diffraction (XRD) and the surface morphology was observed by using scanning electron microscopy (SEM). The in vitro bioactivity and the release of osteo-stimulatory Mg^2+^ ions from the prepared samples were investigated over 7 days of incubation in simulated body fluids (SBF). In vitro cellular analyses revealed the compatibility of the Mg-doped BGs with human osteosarcoma cells (MG-63 cell line). Moreover, the Mg-doped BGs could induce bone nodule formation in vitro and improve the migratory ability of human umbilical vein endothelial cells (HUVECs). In vivo osteogenic capacity was further evaluated by implanting the BG-derived scaffolds into surgically-created critical-size bone defects in rats. Histological and immunohistological observations revealed an appropriate bone regeneration in the animals receiving the glass-based scaffolds after 12 weeks of surgery. In conclusion, our study indicates the effectiveness of the Mg-doped BGs in stimulating osteogenesis in both in vitro and in vivo conditions.

## 1. Introduction

Exploring efficient strategies for treating bone lesions has gained increasing significance in recent years due to the worldwide raise in accidental injuries, obesity, congenital genetic abnormalities, and, more importantly, the aging population [1]. In this regard, the use of transplant materials (e.g., autografts, allografts, and xenografts) has been quite promising; however, several limitations are associated to their extensive usage in the clinic, including the shortage of donors, the risk of immune rejection, and the possibility of zoonoses (disease or infection transmissible from vertebrate animals to humans) when xenografts are used [2]. In order to overcome these barriers, researchers have accomplished successful tissue engineering platforms to fabricate and optimize bone substitutes in the lab towards the clinic [3,4]. Synthetic biomaterials have revealed some important benefits including high reproducibility, easy and unlimited availability, as well as their stable quality, which make them better candidates compared to natural biomaterials [4]. Bioactive glasses (BGs) as a subclass of synthetic bone alternatives have attained great attention in regenerative medicine due to their constructive characteristics in inducing angiogenesis and osteogenesis [5,6,7,8]. Furthermore, the granule/particulate form of these BGs is the most clinically applicable form, which simply fits the defect anatomy due to some advantages including a high surface-to-volume ratio, the ease of handling, and potent hemostatic properties [9]. Bioactive scaffolds, used as a proper environment with a porous temporary supporting structure for the 3D growth of cells/tissues, are commonly available and capable of integrating with bone tissue in order to improve bone regeneration [10]. These properties make scaffolds highly promising bone substitutes with optimized porosity and satisfying mechanical stability in inducing bone regeneration through facilitating cell migration and attachment in a comparable rate to that of natural new bone formation [11]. 

Designing BG scaffolds with novel compositions has continuously progressed to finely tune and optimize their biological and physico-chemical properties. The incorporation of small amounts of therapeutic metal ions into the bioactive scaffold structure has become the most attractive strategy to reach the maximum medical efficiency of the platform [12,13]. Magnesium (Mg), as an alkaline intracellular earth metal with the total content of about 24 g (1 mole) per 70 kg in an adult body mainly located in bones and other soft tissues (e.g., muscles), can be used in the treatment of osteoporosis and bone repair since it is capable of reducing fracture risk and increasing bone density through inhibiting the osteoclast functions as well as promoting the osteoblast activities [14,15]. Furthermore, because 1.11 wt% of dentin and 0.47 wt% of bones consists of Mg^2+^ ions, it plays an essential role in different biological and biochemical processes in the human body including cytoskeletal integrity via the synthesis of proteins and nucleic acids, regulating active calcium transport, and activating phagocytosis as well as skeletal tissue development and bone remodeling [16,17]. 

Thereby, the addition of Mg to BGs has been thought of great importance due to its biocompatibility and applicability in the human body for targeted therapy as well as its key role in bone tissue development; furthermore, MgO can modify and adjust the mechanical, thermal, and physical properties of such silicate-based platforms. In order to improve available treatment strategies, this study focuses on the development of Mg-doped melt-derived BG scaffolds that were implanted in rats to evaluate their bone regenerative potential.

## 2. Materials and Methods

### 2.1. Glass Synthesis and Scaffold Fabrication

The glass (45SiO_2_–3P_2_O_5_–26CaO–15Na_2_O–7MgO–4K_2_O mol.%) was produced by melting and quenching in cold water, as described elsewhere [18]. Briefly, the glass precursors (high-purity powders of SiO_2_, Ca_3_(PO_4_)_2_, CaCO_3_, (MgCO_3_)_4_·Mg(OH)_2_·5H_2_O, Na_2_CO_3,_ and K_2_CO_3_, all purchased from Sigma-Aldrich (Saint-Louis, MO, USA) were carefully weighed by using an analytical balance, homogeneously mixed and heated to 1500 °C (heating rate: 12 °C/min) in a platinum crucible inside an electrically-heated furnace (Nabertherm 1800, Nabertherm GmbH, Lilienthal, Germany). The melt was cast into cold deionized water to obtain a “frit”. The “frit” was pulverized by ball milling, and the glass particles were finally sieved below 32 µm for further analysis and processing. 

BG-derived scaffolds with a trabecular-like 3D architecture mimicking that of cancellous bone were produced by polymer sponge replication [19]. Sacrificial porous polyurethane cuboids (10 mm × 10 mm × 10 mm) were obtained from a panel of open-cell polyurethane (45 ppi) by using a scalpel and soaked in a water-based glass slurry so that the glass particles could coat the foam struts and surface, as described elsewhere [20]. After being dried for 6 h in air, the samples underwent a thermal treatment (950 °C for 3 h inside a Nabertherm Muffle Furnace 1300 L9/11/SKM/P330 (Nabertherm GmbH, Lilienthal, Germany); heating rate: 5 °C/min) to obtain sintered scaffolds replicating the pore/strut architecture of the porous polymeric template.

### 2.2. Characterizations

#### 2.2.1. Bioactivity Assessment in Simulated Body Fluid (SBF)

In order to clarify the bioactivity potential of Mg-doped BGs, a simulated body fluid (SBF) was prepared according to the method described by Kokubo and Takadama [21]. In brief, 150 mg of glass powder was immersed in 100 mL of SBF and incubated at 37 °C in an orbital shaker (KS 4000i control, IKA, Staufen city, Germany) at a constant agitation of 200 rpm for 1, 3, and 7 days. At each time point, the glass powders were collected and rinsed with acetone (Merck, Darmstadt, Germany) to halt any further reaction. The formation of a hydroxyapatite layer on the BG surface was analyzed by using X-ray diffraction (XRD) technique, and the morphology was studied with field-emission scanning electron microscopy (FESEM) (MIRA3, TESCAN, CZ). 

#### 2.2.2. Crystallization Degree Analysis by XRD

The phase composition of the materials produced was determined by X-ray diffraction (XRD) (Explorer GNR, Novara, Italy) before and after incubation in SBF. For this aim, the synthesized samples were introduced to an X-ray powder diffractometer equipped with a monochromatized Cu-Kα radiation (λ =1.54056 Å) in the 2Ɵ range of 20 to 70° with a step size of 0.02°, time per step being 1 s. The crystallization degree, crystallite size, and lattice constants of the crystalline phases were calculated by the Rietveld method by using Profex software (Profex 4.3.5, Open-source package for windows, Nicola Döbelin, Solothurn, Switzerland) [22]. 

#### 2.2.3. pH Variation Measurements 

In order to study the dissolution process of the Mg-doped glasses, pH variations were measured at the end of the incubation timepoints in SBF (1, 3, and 7 days). To this end, the pH values of the solution were monitored by using a universal electronic pH meter (Hanna, Limena, Padova, Italy), which was calibrated according to internal standard operating procedures.

#### 2.2.4. Investigating Ion Release by ICP Analysis

The biological performance of implanted BGs mainly depends on the release of ions from their network into the surrounding biological environment. Accordingly, the release kinetics of ions (K^+^, Na^+^, Si^4+^, Ca^2+^, P^5+^, and Mg^2+^) from the Mg-doped BGs were analyzed by inductively-coupled plasma (ICP) atomic emission spectroscopy (ICP-AES, Spectro Arcos, Kleve, Germany). 

### 2.3. In Vitro Cellular Responses 

#### 2.3.1. Cell Culture 

The response of cells to the BG-derived scaffolds was first evaluated through different in vitro cellular assays, including cell viability, attachment, and migration, as well as osteogenic differentiation. The biological responses were measured after culturing mammalian cells in a media containing ionic dissolution products released from crushed scaffolds, named the conditioned media hereafter. In order to prepare the conditioned media, the samples were first sterilized by UV irradiation for one hour and then soaked (4 mg/mL) in the RPMI-1640 medium supplemented with 10% fetal bovine serum (FBS) and 1% penicillin/streptomycin (P/S).

#### 2.3.2. Viability Assessments by MTT Assay

The effect of ionic dissolution products released from the samples on the proliferation of osteosarcoma cells (MG-63 cell line) was evaluated by using the MTT assay. In brief, a density of 5 × 10^3^ cells was seeded in 96-well plates and incubated in a 5% CO_2_ humidified atmosphere at 37 °C overnight. Thereafter, the media were replaced by conditioned media containing 4 mg/mL of crushed scaffold and incubated for a further 24 h. Then, 10 µL of the 3-(4,5-dimethylthiazol-2-yl)-2,5-diphenyl-2H-tetrazolium bromide (MTT) solution with a final concentration of 0.5 mg/mL was added to each well and incubated for another 4 h at 37 °C. The supernatant of the cell culture wells was gently pulled out and replaced with 100 µL of dimethyl sulfoxide (DMSO) solution (Sigma-Aldrich, Saint-Louis, MO, USA) to dissolve the generated formazan crystals. After incubation in the dark for 20 min, the optical density (OD) of dissolved formazan in each well was recorded at a wavelength of 570 by a microplate reader (Epoch, BioTek, Winooski, VT, USA).

#### 2.3.3. Cell Scratch Migration Assay

The effect of the ionic dissolution product of scaffolds on the migration of human umbilical vein endothelial cells (HUVECs) was assessed using an in vitro scratch assay [23]. Briefly, 1 × 10^4^ HUVECs were seeded into the 24-well plates and cultured with the DMEM-F12 medium supplemented with 5% FBS and 1% P/S for an overnight at 37 °C to achieve a confluent monolayer of the cells. Then, the cell monolayer was scraped by a standard pipet tip (200 µL) in a straight way to generate a scratch. After removing the debris by washing, the condition media (containing 4 mg/mL of crushed scaffold) supplemented with 1% FBS and 1% P/S were added to each well. The level of cell migration was measured via comparing the images captured from time 0 to defined intervals (6, 12, 24, 48, and 72 h) using an inverted microscope (Olympus, Japan). Finally, the data were quantified by using Image J software (NIH, Bethesda, MD, USA).

#### 2.3.4. In Vitro Osteogenesis by Alizarin Red S Staining 

Alizarin Red S staining assay was performed to investigate the in vitro osteogenic capability of the samples. For this purpose, osteosarcoma cells (MG-63 cell line) in a concentration of 6 × 10^4^ cells were plated in T25 cell culture flasks (SPL Life Sciences, Gyeonggi-do, Korea) and incubated in a 5% CO_2_ humidified atmosphere at 37 °C overnight. Then, the RPMI-1640 media were replaced with the conditioned media (containing 4 mg/mL of crushed scaffold) and followed by a further incubation for 14 days. The media were exchanged each 3–4 days once. After finishing the incubation time, all the conditioned media were aspirated, and the cells were carefully washed with PBS (three times) and fixed with a sufficient amount of 10% neutral buffered formalin (NBF) for 30 min. Afterwards, the NBF was removed, and the cells were washed with distilled water. Finally, the cells were stained with Alizarin Red S (Sigma-Aldrich, USA) for 45 min at room temperature in the dark. The amount of generated calcium deposits was detected by optical microscopy observations. 

### 2.4. In Vivo Biocompatibility Study 

#### 2.4.1. Surgical Procedure

**Figure 1:**
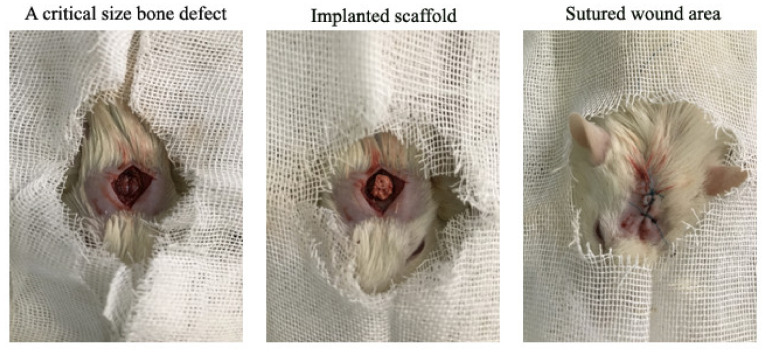
The surgical procedure for creating of critically-sized bone defects and subsequent implanting of the Mg-doped scaffold into rats’ calvaria.

Twenty Wistar rats at 8–10 weeks of age with an average weight of 200–250 g were purchased from Iran University of Medical Sciences, Tehran, Iran. All surgical procedures on the rats were conducted under the ethical IR-approved animal research protocol (IR.IUMS.REC.1389.2014) in accordance with the internationally accepted principles for laboratory animal use [24]. Following an acclimatization period of one week, 20 rats were randomly assigned into the control (4 and 12 weeks) and experimental (4 and 12 weeks) groups. The animals in control groups received no treatment while the rats in the experimental groups were treated with the Mg-doped scaffolds. After anesthetization by 25 mg/kg of ketamine and 5 mg/kg of xylazine, a 6.0 mm midline incision was made on the animals’ calvaria from the nasofrontal area to the external occipital protuberance along the midsagittal suture. Under constant irrigation with sterile saline, a round 6.0 mm diameter segment was detached using a trephine bur. The scaffolds, having a size of about 6 mm due to the volumetric shrinkage occurring upon sintering and post-sintering polishing to achieve an adequate matching with defect anatomy, were then implanted, and the skin edges were sutured by using vicryl cotton 3/0 wires (Ethicon Ltd., Edinburgh, UK), and tetracycline ointment was applied to prohibit infection (Figure 1). The animals were finally transferred into the animal household after recovery, receiving 1 mg/kg of acepromazine to relieve postoperative pain.

#### 2.4.2. Tissue Harvesting and Processing

The animals were sacrificed at 4 and 12 weeks post-implantation, and calvaria samples were harvested and fixed in neutral buffered formalin (NBF) (10%) for 24 h at room temperature. The harvested samples were then exposed to a 10% nitric acid solution (Merck, Darmstadt, Germany) for 24 h in order to remove the bone minerals. Afterwards, the demineralized samples were dehydrated in a series of ethanol solutions with increasing concentrations of 70, 90, 95, and 100%, which was followed by clearing the specimens by xylene and embedding in paraffin. Lastly, serial sections of the samples were cut with a thickness of 5 µm by using a microtome (Leica, Nussloch, Germany).

#### 2.4.3. Histology and Immunohistochemistry Evaluations 

The harvested tissue samples were stained by a commercial H&E staining kit (Sigma-Aldrich, USA) to investigate the biocompatibility and the bone regeneration potential of the implanted Mg-doped BG-derived scaffolds. In addition, immunohistochemistry (IHC) was performed against osteonectin and osteocalcin proteins for 12-week implanted samples to provide detailed information about the bone regeneration capacity of the glasses. In brief, rehydration of the deparaffinized tissue segments was first performed in PBS. This was followed by incubation of the slides in 3% hydrogen peroxide in methanol (Merck, Darmstadt, Germany) for 10 min in order to inactivate the endogenous peroxidase. Antigen unmasking was conducted via treating the samples in citrate buffer in an oven for 10 min. The permeabilization of the slides was performed by their incubation in 0.3% Triton X-100 (Sigma-Aldrich, USA) for 30 min. Then, the slides were incubated with 10% normal goat serum to block non-specific binding, followed by incubation with the primary antibodies including anti-Osteocalcin and anti-Osteonectin monoclonal antibodies (Abcam, UK) at 4 °C overnight. After thrice washing with PBS, the tissues were incubated with goat anti-rabbit-(IgG) antibody conjugated with Alexa-Fluor 488^®^ for 1 h. As the final step, the slides were stained by 4′,6-diamidino-2-phenylindole (DAPI) (Sigma-Aldrich, USA) to stain the nuclei and then visualized with fluorescent microscopy (Olympus, Tokyo, Japan).

### 2.5. Statistical Evaluations

The results of cell viability and migration analyses were statistically assessed by using the nonparametric Kruskal–Wallis test. Statistical significance was considered at probability values of *p* < 0.05 (GraphPad, San Diego, CA, USA). All the experiments were performed three times and all data is presented as the mean ± standard error.

## 3. Results 

### 3.1. Physico-Chemical Characteristics 

#### 3.1.1. XRD Analysis

**Figure 2:**
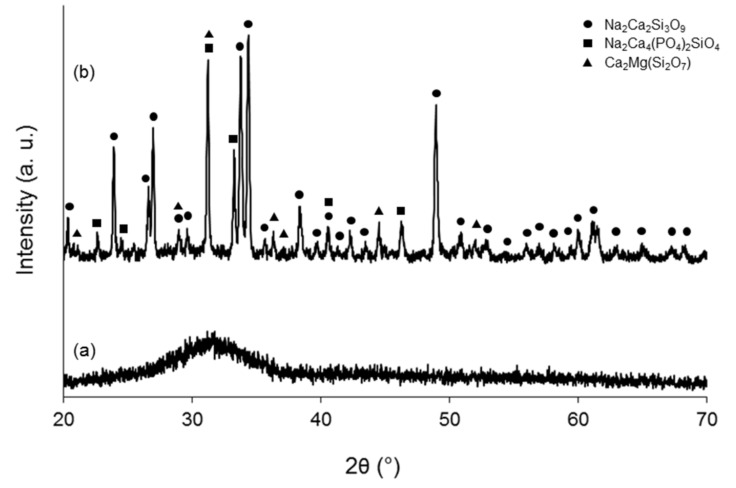
XRD patterns of (**a**) as-quenched glass powder and (**b**) pulverized scaffold (sintered at 950 °C for 3 h) before immersion in SBF.

Figure 2 shows the comparison between XRD patterns of as-quenched glass and pulverized glass-derived scaffold. The broad “bump” in Figure 2a confirms that the starting material was completely amorphous. Upon thermal treatment used for scaffold production (950 °C for 3 h), glass particles undergo sinter-crystallization as confirmed by the development of three crystalline phases (Figure 2b) that were identified as Na_2_Ca_2_Si_3_O_9_ (combeite), Na_2_Ca_4_(PO_4_)_2_SiO_4_ (silicorhenanite), and Ca_2_MgSi_2_O_7_ (akermanite). Therefore, the resulting scaffolds are glass-ceramic materials.

#### 3.1.2. Bioactivity Assessment

**Figure 3:**
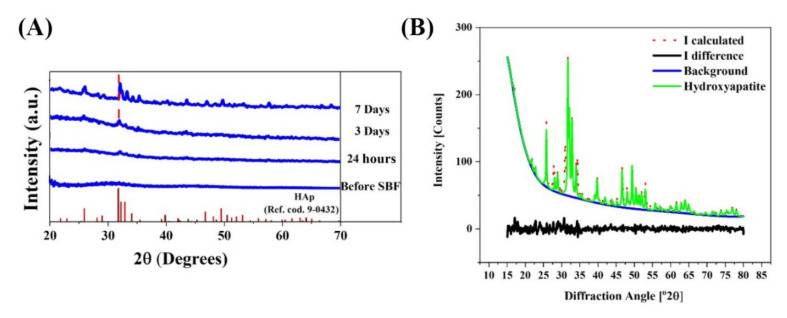
(**A**) X-ray diffraction of the Mg-doped BGs before and after immersion in SBF, which confirms the formation of a HAp layer onto the SBF-immersed samples. (**B**) Rietveld refinement results of the formed HAp after 7 days.

The XRD patterns of the glass powders before and after incubation in SBF are shown in Figure 3a. Besides, the results of the calculation of crystallinity degree, crystallite size, and lattice constants are represented in Table 1. According to the data, the crystallinity degree of glass powders before immersion in SBF is lower than 5%, confirming the amorphous state of the synthesized glass. The phase transformation of glass to hydroxyapatite (HAp) (ICCD ref. code. 9-0432) was detected onto the surface of the SBF-immersed glass; the crystallinity degrees of the sample were 8, 22, and 56% after 1, 3, and 7 days of immersion, revealing that crystallinity increases over time. Moreover, according to the Rietveld refinement results (Figure 3b), the HAp was formed with an almost matched structure with a reference pattern with fitting qualities of R_wp_ = 9.21%, R_exp_ = 3.94%, and GOF = 2.33, which indicate a fairly acceptable refinement. The calculated crystallite sizes of the formed HAp were 12 and 63 nm after 3 and 7 days of immersing, confirming the growth of HAp nano-crystals onto the glass surface during the experiment. The calculated lattice constants of HAp were (a = b = 9.423 and c = 6.883 Å) and (a = b = 9.419 and c = 6.880 Å) after 3 and 7 days of immersion. The lattice constants were almost matched with the ICDD reference (a = 9.418 and c = 6.884 Å). According to the data, a minor reduction of HAp lattice constants was observed in the BGs immersed in SBF for 7 days compared to their counterparts at day 3. 

#### 3.1.3. SEM Observation 

**Figure 4:**
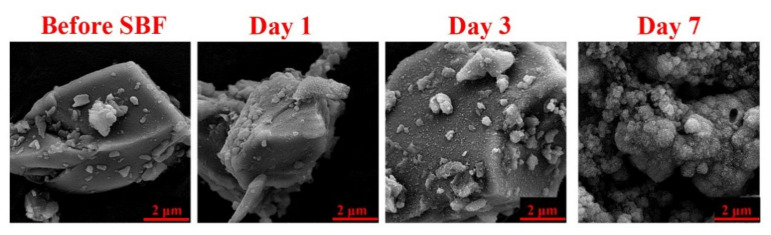
SEM micrographs of the Mg-doped BG particles before and after incubation in SBF (1, 3, and 7 days), which confirm the formation of hydroxyapatite layer over the immersion periods.

Figure 4 shows the morphology and the formation of the HAp layer on the surface of the SBF-immersed Mg-doped BGs. The starting glass particles were recognized to have an irregular shape, and the growth of typical globular HAp agglomerates with cauliflower morphology was clearly detected onto the sample. 

#### 3.1.4. pH Changes 

**Figure 5:**
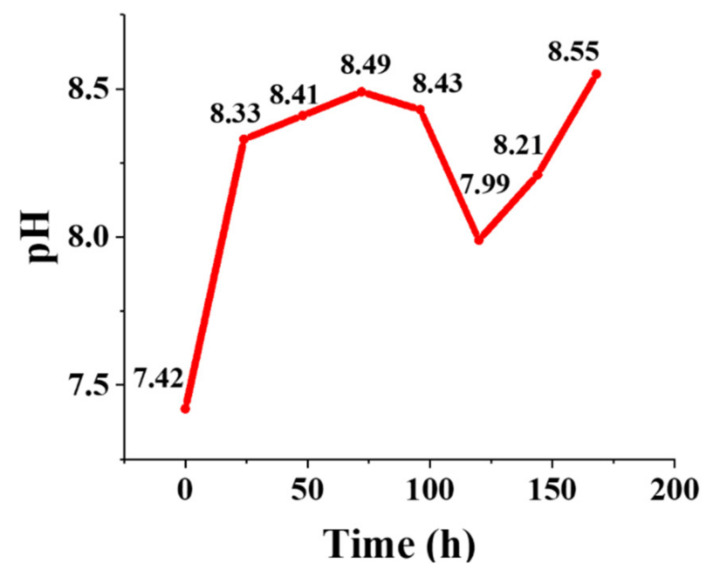
The graph exhibiting the pH variations of the glass-containing medium (SBF) during 7 days post-incubation.

The pH changes in SBF were recorded after the incubation of the Mg-doped BGs during 7 days. As depicted in Figure 5, a sharp increase (from 7.42 to 8.33) was seen during the first 24 h of incubation, which suggests a fast release of alkaline ions in good accordance with the bioactivity mechanism postulated by Hench [25]. The increase in the pH continued more moderately up to day 3, indicating the progressive formation of the HAp layer, and then a decrease was observed until day 5. After that, the pH increased again from 7.99 to 8.55 during days 5 to 7. 

#### 3.1.5. Ion Release

**Figure 6:**
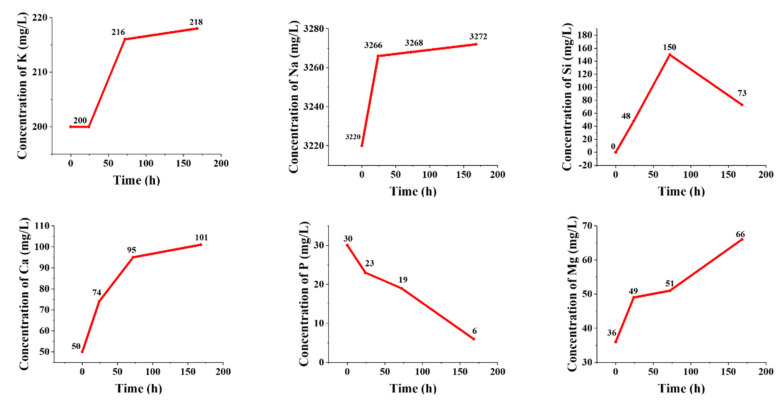
The release kinetic of various ions from the Mg-doped BGs, including K^+^, Na^+^, Si^4+^, Ca^2+^, P^5+^, and Mg^2^+, into the medium (SBF) during 7 days of incubation.

The concentration of ionic species continuously varies during the immersion of bioactive materials in SBF as a result of the dissolution/precipitation phenomena. Figure 6 displays the ion release profile of the Mg-doped BGs over 7 days of incubation in SBF. Moreover, the calculated rates of ion release considering the slope of the curves in the different stages of immersion (linear interpolation) are represented in Table 2. According to these data, the higher release rates of Na^+^, Ca^2+^, and Mg^2+^ ions were related to the first 24 h post-immersion in SBF, while the higher release rates of K^+^ and Si^4+^ ions were detected after 48 h of incubation. The increase of silicon concentration in SBF over the first 2 days corresponds to the release of soluble silica, followed by the formation of the silica gel layer. The release of silicon was then limited when the HAp layer starts to form. The rapid dissolution of the glass within the first day and the decrease of the ion release rate once HAp was formed are confirmed by the concentrations of the Na, Ca, Mg, and K ions. The decrease of the concentration of phosphorous ions is consistent with the formation of HAp, which occurred at the expense of phosphate ion depletion in the solution. Similar ion release kinetics were observed for other melt-derived multicomponent silicate glasses during immersion in SBF [26,27]. 

### 3.2. In Vitro Cellular Responses

#### 3.2.1. Cell Proliferation and In Vitro Osteogenesis

**Figure 7:**
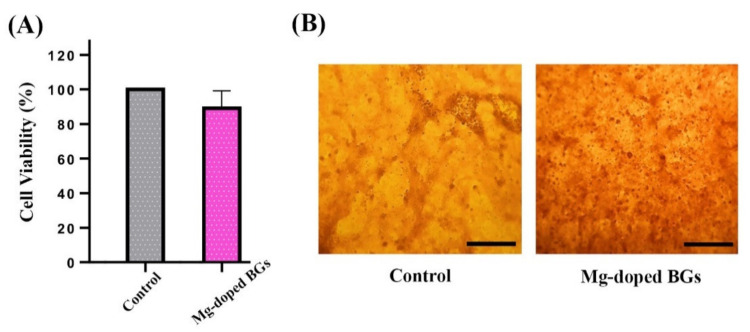
(**A**) The viability assessment of MG-63 cells cultured with the conditioned media containing 4 mg/mL of Mg-doped crushed scaffolds after 24 h incubation. (**B**) Optical microscopic images of alizarin red S stained-cells for (**A**) untreated (control) and (**B**) treated with 4 mg/mL of Mg-doped glass-ceramic at 14 days post-incubation (scale bar: 100 µm).

The potential toxic effects of the Mg-doped crushed scaffolds on osteosarcoma cells (MG-63 cell line) were assessed by a standard cell viability assay (MTT assay). As displayed in Figure 7A, the dissolution product (4 mg/mL) of the glass-ceramic had no significant adverse effect on the cell viability after one day as compared to the untreated cells (control group). The in vitro osteogenic potential of Mg-doped glass-ceramic was determined using an Alizarin Red staining assay. Figure 7B shows the capability of BG dissolution products of stimulating the bone nodule formation after 14 days of incubation. More extracellular calcium deposits are observed in the cells treated with the conditioned media (containing 4 mg/mL of Mg-doped glass-ceramic) in comparison to the untreated cells. 

#### 3.2.2. Cell Migration

**Figure 8:**
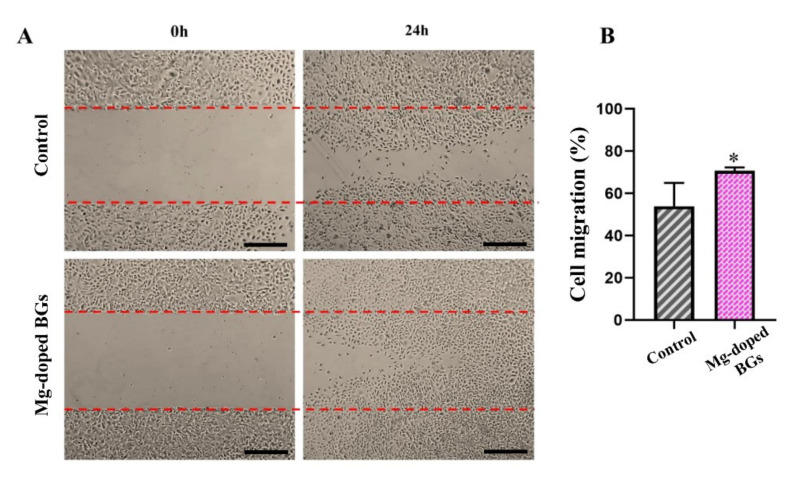
(**A**) The migration rate of HUVECs incubated with or without Mg-doped crushed scaffolds after 0 h and 24 h. (**B**) The graph showing the migratory effects of Mg-doped crushed scaffolds on HUVECs in comparison to the un-treated cells (control). (n = 3, values are represented as mean ± SD, and * *p* < 0.05 for Mg-doped BGs compared to control. Scale bars: 100 µm).

The effects of the crushed scaffolds on the cell movement capability were shown in Figure 8, revealing that HUVECs treated with Mg-doped glass-ceramic had a higher rate of migration in comparison with untreated counterparts (70.8. ± 1.22 vs. 53.85 ± 9.08, respectively) after 24 h of incubation. (*p* < 0.05).

### 3.3. In Vivo Biocompatibility Assessments 

**Figure 9:**
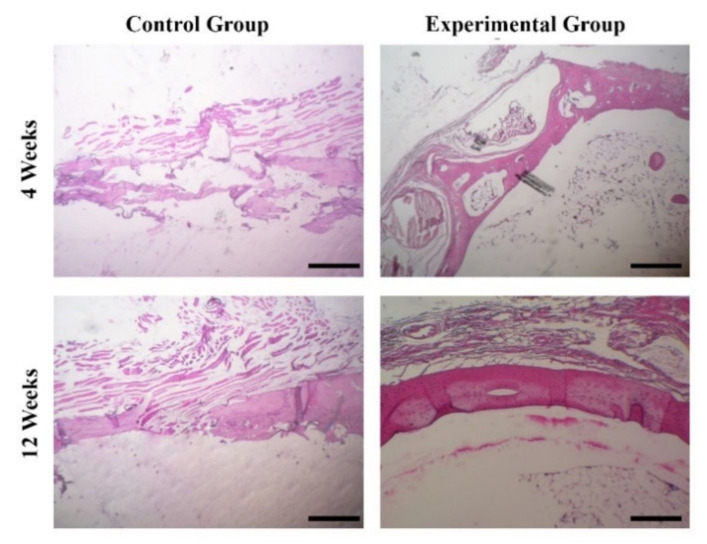
Histological evaluation of the Mg-doped scaffolds implanted into rat calvaria after 4 and 12 weeks. As shown, bone regeneration was clearly observed in the animals receiving the scaffolds.

The in vivo osteogenic potential of the prepared BG-derived scaffolds was initially assessed by H&E staining of the harvested tissues at 4- and 12-weeks post-implantation. As shown in Figure 9, no significant bone regeneration is observed in the control groups (the empty surgical defect) after 4 weeks, while an irregular connective tissue is seen in the samples at 12-weeks post-surgery. Indeed, the osteogenesis occurred in the form of woven bone in the edges and centers of defects treated with the glass-derived scaffolds at 4-week post-implantation. After 12 weeks of implantation, a thicker, newly-formed bone layer is evident over the defects filled with the 3D scaffolds compared to the control groups. Based on Figure 9, it can be claimed that newly-formed, fully mature bone tissue is seen in the center of the defects filled with the Mg-doped glass–ceramic constructs. 

**Figure 10:**
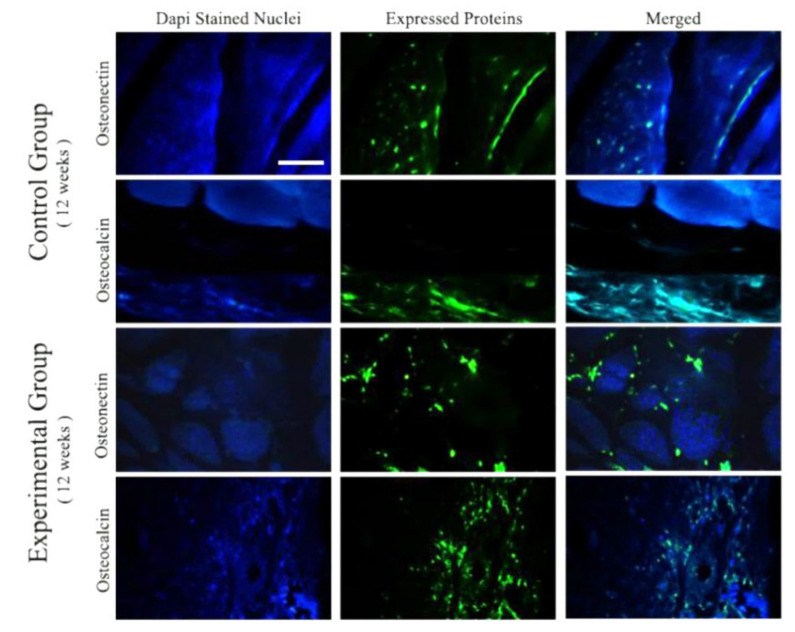
Immunohistochemical staining for the expression evaluation of osteocalcin and osteonectin in the samples after 12 weeks of implantation. As shown, the expression level of both osteogenesis markers is higher in the rats receiving Mg-doped scaffolds, i.e., experimental groups. (Scale bar: 200 µm).

In order to provide more details on in vivo osteogenic potential of the scaffolds, the expression of osteogenic protein markers, including osteonectin and osteocalcin, was detected by the immunostaining technique after 12 weeks of implantation (see Figure 10). As compared to the control groups, the osteogenic proteins were expressed higher in the defects filled with the glass-derived scaffolds, indicating higher osteogenic differentiation and bone mineralization. 

## 4. Discussion

The rules of materials science and engineering are continuously being utilized for the synthesis and preparation of biocompatible substances with the ability to accelerate the repair and regeneration of human damaged tissues. Biocompatible glasses are among the most widely used synthetic materials in both hard and soft tissue engineering strategies [28]; numerous formulations of BGs have been successfully developed by adding therapeutic cations into their basic chemical composition. In this study, we added magnesium (Mg) to a silicate-based BG to prepare an osteogenic tissue substitute, which can be implanted into living systems. Mg is considered a nontoxic element for the human body at a concentration of 10–15 mM [29,30]. This element was previously demonstrated to be an effective element in promoting the proliferation and differentiation of osteoblasts through the activation of the MAPK/ERK signaling pathway [31]. Moreover, Mg can stimulate the proliferation, differentiation, and mineralization of mesenchymal stem cells (MSCs) in a dose-dependent manner through the activation of Notch1 signaling [32]. Pro-angiogenic activity of Mg is regarded as a further added value for its usability in bone tissue engineering. Indeed, Mg can promote angiogenesis through enhancing the production of reactive oxygen species as well as through upregulating VEGF expressions in human endothelial cells [33]. Furthermore, it has been demonstrated that this element may increase new blood vessel formation, leading to improved bone reconstruction in vivo [34,35]. However, it has been reported that exposing Mg to artificial body electrolytes may lead to the formation of Mg(OH)_2_, MgO_2_ oxide, and MgCl_2_. Fortunately, these by-products are excreted or integrated into the natural metabolic process [36]. 

Adding MgO as a glass modifier to the glass network could promote the formation of crystalline phases upon high-temperature thermal treatment [37,38], which was actually confirmed by the formation of akermanite. Figure 2 shows the XRD patterns of the BGs particles and the pulverized scaffolds after sinter-crystallization at 950 °C. In the basic BG, only a broad hump in the range of 25–35° is observed, confirming the amorphous nature of the glass. Sodium calcium silicate (Na_2_Ca_2_(Si_3_O_9_, combeite, ICCD ref. cod. 01-075-1686, rhombohedral), sodium calcium phosphosilicate (Na_2_Ca_4_(PO_4_)_2_SiO_4_, silicorhenanite, ICCD ref. cod. 00-033-1229), and calcium magnesium silicate (Ca_2_MgSi_2_O_7_, akermanite, ICCD ref. cod. 00-079-2425, tetragonal) were detected after heat treatment at 950 °C. Combeite and silicorhenanite are the same crystalline phases that develop in sintered 45S5 Bioglass^®^ [39], which has been in clinical use for 30 years to regenerate bone; akermanite was also reported to promote new bone formation [40,41]. 

Figure 3 shows the results of phase transformation of BGs powders to HAp during the immersion experiments in SBF. According to the results, HAp formed after 1 day of immersion, and its crystallinity increased to about 56% after 7 days of immersion. Interestingly, the lattice constants of HAp were changing during the immersion steps, which can be related to the progressive growth of HAp crystal size and the doping of some release elements to its structure [42]. 

Figure 4 gives detailed information on the shape and surface morphology of the synthesized glass samples. In addition, the progressive formation of a HAp layer onto the Mg-doped BGs can be detected over 7 days of the glass immersion in SBF, which is in line with previously published data [43] and ISO 23,317 (Implants for surgery) [44]. It has been reported that the incorporation of Mg into glasses does not suppress the formation of the surface HAp layer [45,46] but may cause a delay in bioactivity. There are two main reasons behind this phenomenon, which are related to (I) the decrease of the solubility of the glass due to stronger Mg–O chemical bonds than Ca–O bonds and (II) the reduced rate of formation of a more stable apatite phase as a result of Mg leaching to SBF [47,48]. 

The pH changes of the glass-containing SBF are depicted in Figure 5. A sharp increase can be detected in the medium in the early hours after incubation. This increase is attributed to partial dissolution at the BG surfaces due to the high reactivity of this kind of biomaterial [49]. Indeed, an ion interchange occurs between Na^+^/K^+^/Ca^2+^/Mg^2+^ from the glass and H_3_O^+^ from the SBF in the early stages. The process supports the formation of apatite nuclei on the silanol groups on the BG surface, which was previously well-documented [50]. It is worth mentioning that prior studies have revealed that Mg^2+^ ions may concentrate onto the glass surface and change the thermodynamic variables, leading to lower nucleation and crystal growth rates of HAp from its precursor, the amorphous calcium phosphate layer [46].

The release profiles of the elements, including Si, Ca, Na, P, K, and Mg, released from the synthesized glasses into SBF were quantified by ICP analysis and are depicted in Figure 6. As expected, a burst release for alkaline ions was observed during the first hours (up to 24 h) of incubation, while the release rates decreased by day 3. In detail, the highest release rate of Na^+^ (from 3220 to 3266 ppm), Ca^2+^ (from 50 to 74 ppm), and Mg^2+^ (from 36 to 49 ppm) was observed in the first 24 h of incubation, while the highest release rate of K^+^ (from 200 to 216) and Si^4+^ (from 48 to 150 ppm) was observed over the period of 24–72 h. Furthermore, according to the data, P^5+^ ions were depleted from the SBF at all times of the incubation process. The highest release rate of P^5+^ ions were also related to the first 24 h of incubation (from 30 to 23 ppm). The decreasing rate of ion release may be due to the saturation of SBF and the formation of a surface HAp layer. Overall, the ion release trends are consistent with pH measurements. The highest amounts of the measured Si^4+^, Ca^2+^, Na^+^, P^5+^, K^+^, and Mg^2+^ ions released from the samples were 150, 101, 3272, 30, 218, and 66 ppm from day 1 to 7. This sustained release is a promising issue in bone tissue engineering applications, as the slow release of Mg^2+^ may improve the bone marrow-derived mesenchymal stem cell (BMSC) proliferation and osteoblastic activity through the PI3K/Akt/GSK3β/β-catenin signal pathway in vitro [51].

BGs are generally regarded as biocompatible materials with living systems (e.g., mammalian cells). The result of MTT assay (Figure 7A) confirms the lack of adverse effects of the Mg-doped crushed scaffolds on osteosarcoma cells (MG-63 cell line). A slight decrease in the cell proliferation rate of the experimental group may be attributed to the potential effects of BGs in the sudden and sharp increase of the environmental pH [52]. This effect, i.e., the sharp increase of pH caused by the ion release from the BGs, was mentioned to be greatly moderate in the in vivo situation thanks to the high volume of body fluids (e.g., blood) [53]. Figure 7B illustrates the osteogenic capacity of the crushed scaffolds in vitro. As can be seen, the Mg-doped BGs could enhance the bone nodule formation, i.e., calcium deposition, in the osteoblastic cells (MG-63 cell lines) after 14 days. This finding is in agreement with previously reported studies which state that the release of therapeutic ions from BG structure into the surrounding biological environment can stimulate bone regeneration [54]. It should be pointed out that Mg^2+^-containing glasses were previously demonstrated to be effective in the osteogenic differentiation of mesenchymal stem cells (MSCs) [55], which make a strong concept for their utilization in bone regenerative strategies.

In addition to enhancing osteogenesis, the release of specific ions (e.g., Si^4+^ and Ca^2+^) from the BG structure into the biological environment can trigger other biological phenomena, including angiogenesis. In our study, the Mg-doped BGs (4 mg/mL) could enhance the migratory potential of HUVECs as compared with the control group during 24 h post-incubation (Figure 8). This result is in line with previously published studies, which indicate that BGs can stimulate the motility of endothelial cells (e.g., HUVECs) in vitro [8]. 

The Mg-doped glass-derived scaffolds were implanted into critical-sized bone defects in rats in order to investigate their biocompatibility and in vivo osteogenic capacity. It was predictable that the BGs cause no harmful side effects (e.g., chronic inflammation) in the animals’ body as to the selected composition. In fact, the reactive surface of BGs is the main reason for inhibiting the formation of a fibrous capsule around glass-based implants in vivo (a hallmark of a chronic inflammatory response) and facilitating regeneration of tissue [56]. In addition, histology observations (Figure 9) confirmed that the Mg-doped BG-based scaffolds can support bone tissue engineering. These outcomes are in line with previously performed in vivo studies that introduce BGs as osteoinductive materials [57]. It has been well understood that osteocalcin (OC), a bone-specific protein, is produced by osteoblasts and represents the most abundant non-collagenous polypeptide of bone matrix [58]. More importantly, this peptide is regarded a good indicator for osteogenic maturation. Another non-collagenous protein, osteonectin (ON), plays a role in bone development and is known for its affinity for HAp and collagen [59]. Therefore, we evaluated the expression levels of these proteins in the implanted sites (Figure 10). As can be seen, higher amounts of both the osteogenic proteins were expressed in the experimental groups as compared to the control groups. It can be concluded that the implanted glass-derived scaffolds could encourage bone regeneration at the molecular levels at 12 weeks post-surgery.

## 5. Conclusions

Magnesium (Mg)-doped BGs were successfully synthesized and then used to fabricate 3D porous scaffolds by using the sponge replica method. The samples showed a glassy nature after the production and were able to promote HAp formation (bioactivity) post-immersion in SBF. The release of Mg^2+^ ions from the glasses successfully occurred over 7 days of incubation in SBF. The Mg-doped BGs caused no toxicity towards osteoblastic cells and could enhance bone nodule formation in vitro. Moreover, the glass samples could induce the migration of human endothelial cells, which might be regarded as preliminary proof of their pro-angiogenic capacity. In vivo implantation of glass-derived porous scaffolds led to enhanced bone regeneration in rats at 4- and 12-weeks post-surgery. All in all, it can be stated that Mg-doped BGs can be considered a suitable substitution biomaterial for regenerating injured bones (e.g., flat cranial bones), and the promising results reported in this study motivate further investigations. 

## Figures and Tables

**Table 1:** The results of the calculation of crystallinity degree, crystallite size, and lattice constants of the formed hydroxyapatite onto the Mg-doped BGs.

Sample	Crystallinity(%)				Crystallite Size (nm)		a (=b) (Å)		c (Å)	
Days	0	1	3	7	3	7	3	7	3	7
Glass powder	<5	8	22	56	12	63	9.423	9.419	6.883	6.880
ICCD reference	-	-	-	-	-	-	9.418		6.884	

**Table 2:** The calculated rates of ion release from the Mg-doped BGs.

Time (h)	Ion Release Rate (mg/L h)					
	K^+^	Na^+^	Si^4+^	Ca^2+^	P^5+^	Mg^2+^
0–24	0	1.92	2.00	1	−0.29	0.54
24–72	0.33	0.04	2.12	0.44	−0.08	0.04
72–168	0.02	0.03	−0.80	0.06	−0.14	0.16

## Data Availability

Data available in the study.
